# Mechanical stress activates neurites and somata of myenteric neurons

**DOI:** 10.3389/fncel.2015.00342

**Published:** 2015-09-15

**Authors:** Eva M. Kugler, Klaus Michel, Florian Zeller, Ihsan E. Demir, Güralp O. Ceyhan, Michael Schemann, Gemma Mazzuoli-Weber

**Affiliations:** ^1^Human Biology, Technische Universitaet MuenchenFreising, Germany; ^2^Department of Surgery, Klinikum FreisingFreising, Germany; ^3^Department of Surgery, Klinikum Rechts der Isar, Technische Universitaet MuenchenMunich, Germany

**Keywords:** enteric nervous system, myenteric neuron, mechanosensor, multifunctional, von Frey hair

## Abstract

The particular location of myenteric neurons, sandwiched between the 2 muscle layers of the gut, implies that their somata and neurites undergo mechanical stress during gastrointestinal motility. Existence of mechanosensitive enteric neurons (MEN) is undoubted but many of their basic features remain to be studied. In this study, we used ultra-fast neuroimaging to record activity of primary cultured myenteric neurons of guinea pig and human intestine after von Frey hair evoked deformation of neurites and somata. Independent component analysis was applied to reconstruct neuronal morphology and follow neuronal signals. Of the cultured neurons 45% (114 out of 256, 30 guinea pigs) responded to neurite probing with a burst spike frequency of 13.4 Hz. Action potentials generated at the stimulation site invaded the soma and other neurites. Mechanosensitive sites were expressed across large areas of neurites. Many mechanosensitive neurites appeared to have afferent and efferent functions as those that responded to deformation also conducted spikes coming from the soma. Mechanosensitive neurites were also activated by nicotine application. This supported the concept of multifunctional MEN. 14% of the neurons (13 out of 96, 18 guinea pigs) responded to soma deformation with burst spike discharge of 17.9 Hz. Firing of MEN adapted rapidly (RAMEN), slowly (SAMEN), or ultra-slowly (USAMEN). The majority of MEN showed SAMEN behavior although significantly more RAMEN occurred after neurite probing. Cultured myenteric neurons from human intestine had similar properties. Compared to MEN, dorsal root ganglion neurons were activated by neurite but not by soma deformation with slow adaptation of firing. We demonstrated that MEN exhibit specific features very likely reflecting adaptation to their specialized functions in the gut.

## Introduction

Mechanosensitivity plays a key role in a variety of reflexes in the intestines that constantly contract and relax. Reacting to external environmental changes and modulating gastrointestinal motility in response to different meals are examples of situations that require an adequate response to mechanical stimuli in the gut. The reflex circuits are made up of neurons in the enteric nervous system (ENS), which is a truly autonomous nervous system in the gut wall (Bayliss and Starling, 1899; Gershon, 1981). The ENS consist of two ganglionated plexus: the submucous plexus located underneath the epithelial lining and the myenteric plexus between the two smooth muscle layers.

It has been shown very early that distension of the gut wall triggers a nerve mediated reflex—the so called peristaltic reflex (Bayliss and Starling, 1899). This reflex consists of spatially coordinated muscle contraction and inhibition which guarantees the proximal to distal transit of intraluminal content. It seems plausible that the neuronal mechanosensors are located in the myenteric plexus because this plexus is embedded between the two muscle layers and therefore strategically positioned to sense deformation and control motility. Both, the cell bodies and neurites of myenteric neurons are continuously exposed to deformation during muscle movements (Gabella and Trigg, 1984; Mazzuoli and Schemann, 2009). Indeed, myenteric neurons encode mechanical stimuli to initiate the peristaltic reflex because distension evoked contractile activity is impaired in intestinal segments freed of the myenteric plexus (Magnus, 1904). On the other hand peristaltic reflex activity remains after removal of the mucosa and submucosal plexus, again showing that the relevant neural circuitry lie in the myenteric plexus (Spencer et al., 2003). Mechanosensitive enteric neurons (MEN) have been described by intra and extracellular recording techniques as well as by neuroimaging with voltage sensitive dyes in the myenteric plexus (Mayer and Wood, 1975; Kunze et al., 1998; Weber et al., 2001; Spencer and Smith, 2004; Mao et al., 2006; Mazzuoli and Schemann, 2009, 2012). A specialized group of myenteric neurons, referred to as intrinsic primary afferent neurons (IPANs), has long been suggested to be the only enteric sensory neurons (Bornstein, 1994; Furness et al., 1998, 2004; Bertrand and Thomas, 2004; Gershon, 2005). IPANs in the guinea pig ileum have characteristic features: they show slow after-spike hyperpolarization (AH), lack fast excitatory synaptic input and express the calcium binding protein calbindin. It is these neurons which responded to sustained distension of the gut wall and behave like slowly adapting mechanosensors (Kunze et al., 1998; Quinson et al., 2001; Clerc and Furness, 2004). This concept has been recently expanded by the identification of MEN others than IPANs (Spencer and Smith, 2004; Smith et al., 2007; Mazzuoli and Schemann, 2009, 2012). Our group recently identified multifunctional MEN, referred to as rapidly adapting mechanosensitive enteric neurons (RAMEN) and slowly adapting mechanosensitive enteric neurons (SAMEN), which respond to deformation with spike discharge (Mazzuoli and Schemann, 2009, 2012). RAMEN were described in intact muscle-myenteric plexus preparations of guinea pig ileum and mouse colon (Mazzuoli and Schemann, 2009, 2012). Interestingly, RAMEN share common features in different regions and species (Mazzuoli and Schemann, 2009, 2012) whereas IPANs appear to be rather unique for the myenteric plexus of the guinea pig ileum. Moreover, RAMEN directly responded to ganglion deformation as their response was unaltered after muscle paralysis or blockade of synaptic transmission (Mazzuoli and Schemann, 2009).

Attempts to study mechanosensitivity of soma and neurites have been only performed in guinea pig myenteric IPANs. In these neurons, neurite deformation enhanced while soma compression inhibited spike discharge (Kunze et al., 2000). These experiments have been performed in intact tissue by probing interganglionic fiber tracts or ganglionic regions with von Frey hairs. Similar experiments in intact tissue have been performed on IPANs in the mouse intestine probing myenteric ganglia with von Frey hairs (Mao et al., 2006). Mechanosensitive interneurons with electrophysiological behavior distinct from IPANs respond to circumferential and/or longitudinal stretch of the gut wall (Spencer and Smith, 2004). All electrophysiological studies on MEN performed so far have some principle limitations that are inherent to the experimental design. The response patterns after a targeted probing of neurites cannot be studied in intact gut preparations because of the large number of nerve fibers running together in ganglia and interganglionic fiber tracts. It is therefore not feasible to follow action potentials along single neurites and to record their propagation into the soma. In addition, ganglia are embedded in muscle and connective tissue and surrounded by other cells. While this has been an advantage to study the involvement of non-neuronal cells in mechanotransduction, it prevented recordings of direct mechanosensitive responses in MEN.

We therefore studied the behavior of primary cultured mechanosensitive myenteric neurons. This allowed us to perform targeted mechanical stimulation of a single soma and neurite with an ultra-fine carbon fiber which served as a von Frey hair. With this approach mechanical stress was locally applied to a defined region. Furthermore, we used a neuroimaging technique together with the voltage sensitive dye Di-8-ANEPPS to record spike discharge. This technique in combination with an independent component analysis (ICA) allowed us to detect signals from a single soma and neurite. The origin of the electrical signal could be studied and followed from the site of mechanical stimulation to the soma and to other neurites. The responses obtained in mechanosensitive myenteric neurons were compared with those recorded in dorsal root ganglion (DRG) neurons. DRG nerve endings have with well-established mechanosensitive properties transmitting information from the periphery to higher centers.

## Materials and methods

### Ethical approval

All guinea pig work was conducted according to the German guidelines for animal care and welfare (Deutsches Tierschutzgesetz) and approved by the Bavarian state ethics committee (Regierung Oberbayern, which serves as the Institutional Care and Use Committee for the Technische Universitaet Muenchen) according to §4 and §11 Deutsches Tierschutzgesetz under the reference number 32-568-2.

Procedures for the work with human samples were approved by the ethical committee of the Technische Universitaet Muenchen (1746/07; informed consent was obtained from all patients).

### Culture technique

Myenteric neurons from guinea pig small intestine and human intestinal tissue samples were cultured as previously described (Vanden Berghe et al., 2000; Kugler et al., 2012; Schemann et al., 2012). Briefly, 68 male guinea pigs (Dunkin Hartley, Harlan GmbH, Borchen, Germany) were killed by cervical dislocation followed by exsanguination (approved by the local animal ethical committee and according to the German guidelines for animal protection and animal welfare). A 10 cm piece of the small intestine was quickly removed. The myenteric plexus together with longitudinal muscle and serosa was stripped with fine forceps.

For human myenteric plexus preparations human tissue samples of large (9 ×) and small intestine (9 ×) were obtained of 18 patients (9 × male, 9 × female, 68 ±15 years) undergoing surgery at the medical clinics of Freising and Rechts der Isar in Munich (Germany). Samples were taken from macroscopically unaffected areas. Diagnoses that led to surgery were carcinoma (13), polyp (1), endometriosis (1), sigma diverticulitis (1), ileostoma (1), and sigma stenosis (1). After surgery, tissue samples were placed in ice-cold oxygenated sterile Krebs solution and immediately transferred to the laboratory. Tissues were dissected in ice-cold oxygenated sterile Krebs solution containing (in mM): 121 NaCl, 6 KCl, 12 Glucose, 14 NaHCO_3_, 1 NaH_2_PO_4_, 1 MgCl_2_^*^ 6 H_2_O, 3 CaCl_2_^*^ 2 H_2_O; in order to obtain longitudinal muscle—myenteric plexus (LMMP) preparations.

Further preparations of human and guinea pig tissue samples were performed under sterile conditions. The LMMP preparations of both species were cut in ~1 × 1 mm pieces and digested for 20–70 min in an enzymatic solution containing 0.9 mg mL-1 protease type I from bovine pancreas (Sigma-Aldrich, Steinheim, Germany), 1.2 mg mL-1 collagenase type II from clostridium histolyticum (Gibco, Karlsruhe, Germany) and 3.7 mg mL-1 bovine serum albumine fraction V (Serva, Heidelberg, Germany) at 37°C. Enzymatic digestion was followed by centrifugation (1000 rpm, 5 min, 3 ×) and washing steps with ice-cold sterile Krebs solution. The obtained pellet was dissolved in medium M199 (Gibco). From this suspension ganglia were picked under a stereomicroscope (Leica DMIL, 4 × objectives with phase contrast) and 2–5 dishes (ibidi μ-dish 35 mm with ibiTreat-coating, Ibidi, Martinsried, Germany) were inoculated with cell suspension. Myenteric ganglia were incubated in medium M199 supplemented with 10% FBS (Gibco), 50–100 ng/mL mouse nerve growth factor 7S (Alomone labs, Jerusalem, Israel), 5 mg/mL Glucose, 100 U/mL Pencillin, 100 μg/mL Streptomycin (Gibco) and 2 μM arabinose-C-furanoside (Sigma-Aldrich).

Primary cultures of guinea pig DRG neurons were performed as previously published (Buhner et al., 2014). The spinal column of the guinea pig was isolated and then placed into sterile, ice-cold Krebs solution. The DRG were isolated under a stereomicroscope (Leica DMIL). The DRG were washed 3 times in fresh solution and collected in a small glass container, cut into small pieces with scissors and digested in the same enzymatic solution as above. After 40–50 min enzymatic digestion at 37°C tissue samples were washed by centrifugation (1000 rpm, 5 min, 3 ×) with ice-cold Krebs solution. Thereafter the pellet was suspended in 600–1200 μL cold culture medium (M199) supplements as mentioned above. Each culture dish was inoculated with 300 μL ganglia suspension; 2–4 culture dishes can be inoculated from the DRG of one guinea pig.

The medium was changed every 2–3 days. At the first day after the isolation the cultured neurons adhere to the bottom of the dish. After 48–72 h the myenteric neurons tend to reorganize themselves in ganglion-like structures defined as cluster (2–30 neurons) forming a network. The DRG cultured neurons adhere to the bottom of the dish either as single cells or in clusters of 2–20 neurons. After 48–72 h the neurite outgrowth starts. The cultures were grown at least for 7 days to get long neurites before performing an experiment.

For the recordings the dishes were placed in a custom-made culture dish holder and continuously superfused with 37°C Hepes modified Krebs solution (pH = 7.4) containing (in mM): 1 MgCl_2_^*^ 6 H_2_O, 2 CaCl_2_^*^ 2 H_2_O, 150 NaCl, 5 KCl, 10 Glucose, 10 Hepes.

### Multisite optical recording technique

To detect electrical signals in cultured myenteric neurons we used an ultrafast imaging technique with the fluorescent voltage sensitive dye 1-(3-sulfanato-propyl)-4-[β-[2-(di-n-octylamino)-6-naphtyl] vinyl]pyridinium betaine (Di-8-ANEPPS) (Michel et al., 2011). To stain the cultures they were incubated with 10 μM Di-8-ANEPPS for 10–20 min at room temperature in the dark. It has been shown that Di-8-ANEPPS stimulates potassium activated (BK(Ca)) channels (Wu et al., 2008). However, we have no evidence that any Di-8-ANEPPS pharmacology compromises electrophysiological behavior of enteric neurons. This conclusion is based on intracellular recordings before and after Di-8-ANEPPS staining (Neunlist et al., 1999). The culture dish was mounted onto an inverted epifluorescence microscope (Zeiss Axio Observer.A1; Munich, Germany). To detect the signals of Di-8-ANEPPS the microscope was equipped with a modified Cy3 filter set (545 ± 15 nm excitation, 565 nm dichroic mirror, 580 nm barrier; AHF Analysentechnik, Tübingen, Germany) and excited by a green LED (LE T S2W, Osram, Munich, Germany). Signal acquisition and processing was performed with the Neuroplex 9.1.1 software (RedShirt Imaging, Decatur, GA, USA). The changes of the membrane potential are linearly related to the relative changes in the fluorescence (ΔF/F) that is measured by a cooled charge coupled device (CCD) camera (80 × 80 pixels, RedShirt Imaging) (Neunlist et al., 1999). Optical signals of voltage sensitive dye were recorded with a frame rate of 1–10 kHz, which enables the detection of action potentials. Dye bleaching was avoided using relatively short exposures not exceeding 5 s, that were still sufficient to reveal mechanosensitive response (Neunlist et al., 1999; Mazzuoli and Schemann, 2009; Michel et al., 2011). With an oil immersion × 100 objective (*NA* = 1.35, Olympus, Hamburg, Germany) the spatial resolution of the CCD camera is 4.68 μm^2^/pixel. With an average area of 219 μm^2^ per neuron (own data, see Results Section) several pixels recorded activity over single neurons. For the analysis the optical/electrical signals and images of neurons clusters were overlaid (Michel et al., 2005). It was thereby possible to detect signals of each individual soma as well as to track the signals along the neurites.

For the detection of action potential firing with Di-8-ANEPPS in response to mechanical stimulation a recording time of 2000 ms at 1 kHz and an x100 objective were used. To calculate the conduction velocity recordings of 2000 ms at 10 kHz with an oil immersion x40 objective were performed. Nicotine (Sigma-Aldrich) 100 μM was applied by pressure ejection from a glass pipette with fine opening (~10 μm) positioned ~500 μm from the cell cluster in order to reveal activation of soma and neurites before applying mechanical stress and to test viability of the neurons. Acquisitions without any stimulus were performed to check for spontaneously active neurons.

Pictures of the clusters were taken with a high spatial resolution camera (Axiocam ICm1; Zeiss).

### Mechanical stimulation

Targeted deformation of soma and neurites was performed with an ultra-fine von Frey hair. A single carbon fiber (Ø 8 μm, surface area 50 μm^2^; Conrad, Hirschau, Germany) was inserted into a glass capillary (0.58 × 1.00 × 100 mm, Science Products, Hofheim, Germany). This capillary was then pulled (Puller P87, Sutter instrument). The carbon fiber extended the fine tip of the glass pipette by 400–900 μm. The position of this ultra-fine von Frey hair was controlled by a motorized Piezo-driven micromanipulator (PM-10 with DC-3K, Märzhäuser, Wetzlar, Germany) that allows for fine movements (step sizes 1–10 μm) with a fixed speed of 100 mm/s. The Piezo-driven manipulator exerted the maximal force within less than 1 ms. The carbon fiber was pressed onto the neurons at an angle of 74 ± 4° (*n* = 8) for a 10 μm step size leading to a the maximal exerting force of 0.4 ± 0.05 mN. The force was measured by pressing the carbon fiber with a 10 μm step onto a force transducer (MLT1030/a; AD instruments Ltd, Oxford, UK), which was calibrated before with water drops of different weights. Neurons were probed with the von Frey hair during the entire recording period (2–10 s).

### Nerve fiber stimulation and local application of tetrodotoxin

To block axonal and soma action potentials we applied the neurotoxin tetrodotoxin (TTX). A stock solution of TTX (1 mM; Biozol Diagnostica, Eching, Germany) was prepared. TTX was diluted to a final concentration of 10 μM with the same Hepes modified Krebs solution as superfused during the experiment. To achieve only a local blockade of the neurites we applied TTX via a fine glass pipette with an opening of ~3 μm positioned ~5 μm away from the neurite that we intended to block. Thus, TTX was applied only onto a small area of the neurite proximal (closer to the soma) to the mechanical stimulation site. To visualize this area we applied with the same glass pipette a small quantity of fast green (data not shown). TTX was applied 3 times for 60 s. As a control for local blockade we electrically stimulated the neurite just next to the TTX pipette with a microelectrode made out of a carbon fiber (electrode was further away from the soma). The stimulation parameters were: 600 μs pulse duration at 50 μA. Electrical pulses through the microelectrode as well as mechanical stimuli were applied before, during and 10 min after TTX application.

### Immunohistochemistry

Immediately after the neuroimaging experiments, the cultured neurons were fixed overnight at 4°C in a solution containing 4% paraformaldehyde and 0.2% picric acid in 0.1 mol/L phosphate buffer and then washed (3 × 10 min) in phosphate buffer. A blocking serum with phosphate-buffered saline (PBS) containing 0.5% Triton X-100, 0.1% NaN3 and 4% horse serum was incubated for 1 h at room temperature. This was followed by 12 h incubation with the primary antibody and 1.5 h with the secondary antibody diluted with blocking serum. As primary antibodies we used rabbit anti-calbindin (Calbindin; 1:1000; Chemicon, Limburg, Germany) and sheep anti-human protein gene product 9.5 (PGP 9.5; 1:20,000; The Binding Site, Birmingham, UK). As secondary antibodies we used donkey anti-rabbit conjugated to 7-amino-4-indodicarbocyanin (Cy5; 1:200; Dianova, Hamburg, Germany), donkey anti-sheep conjugated to CF 488 (Cy2; 1:200; Biotium, Hayward, CA, USA). After immunohistochemistry the cultured neurons were treated for 1.5 h with 1 mM CuSO4 in 50 mM ammonium acetate buffer to reduce autofluorescence of the cells. Finally the cells were washed in PBS and mounted with a solution of PBS (pH 7.0) and 0.1% NaN3 containing 80% glycerol. The preparations were examined with an epifluorescence microscope (Olympus) equipped with appropriate filter blocks. Images were acquired with a video camera (F-View II, Olympus) connected to a computer and controlled by the “cell^p^” software (Olympus).

### Data analysis and statistics

**Figure 1:**
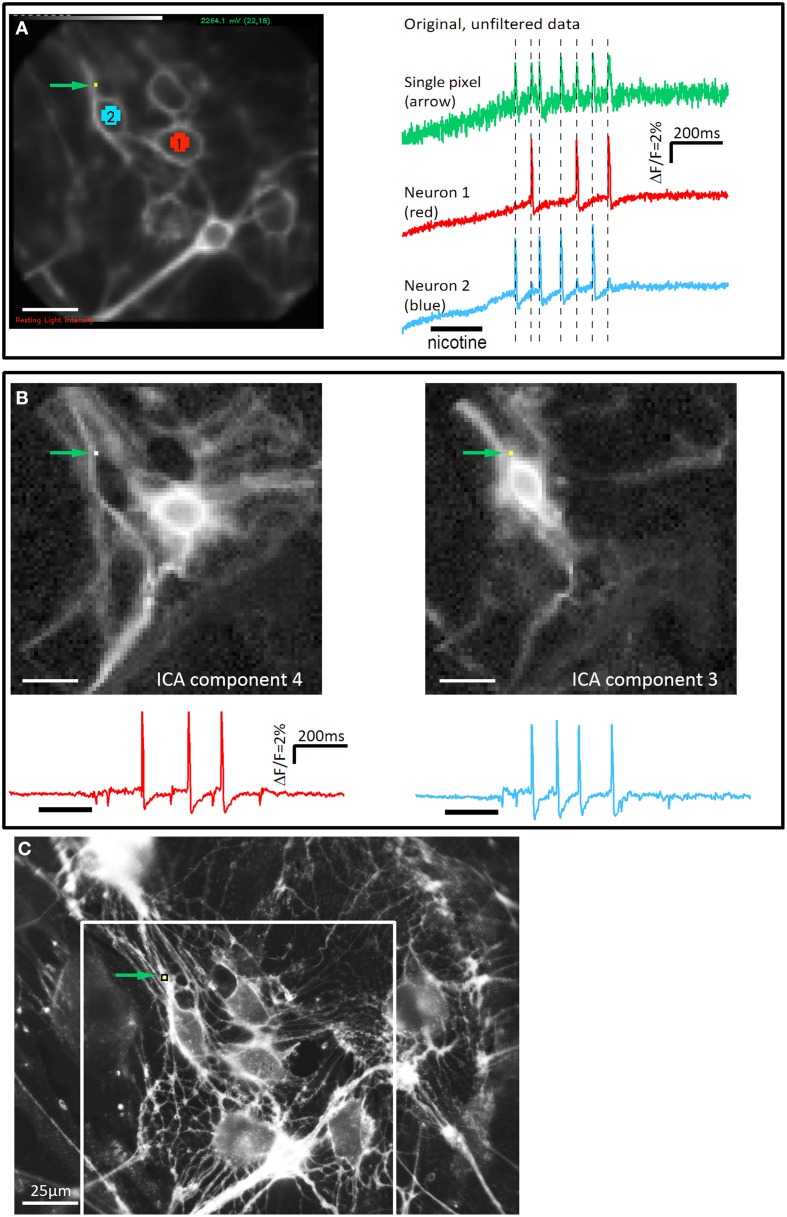
**The independent component analysis is able to separate signals from two neuronal structures on a single pixel. (A)** The picture on the left side illustrates a cluster of cultured guinea pig myenteric neurons taken with the high speed camera (80 × 80 pixels). Pixels on two neurons (neuron 1: red, neuron 2: blue) and a single pixel in the upper left corner (green arrow) are marked. On the right side the corresponding signals from these areas are shown. Nicotine was applied for 200 ms as indicated by the black bar below the traces. Both neurons show a discharge of spikes with different patterns (neuron 1: 3 spikes, neuron 2: 4 spikes). The single pixel shows 7 spikes which exactly coincides with the spikes from neuron 1 and neuron 2 (illustrated by broken lines). The signals of this pixel represent the mixed responses from neurons 1 and 2. **(B)** The complete dataset was analyzed with the ICA method. The discharge pattern from the extracted ICA components 3 and 4 matched the signals from neuron 1 and neuron 2, respectively. In addition, the corresponding images show that the same pixel, which shows the mixed signal in the original data, detects signals from the two neurons (green arrow). **(C)** High resolution picture of the Di-8-ANEPPS stained neurons in the same region as in **A**. Brightness of the picture was increased to show fine neurites more clearly. The field of view of the high speed camera is marked with a white rectangle. Even in this high resolution picture it would not be possible to assign the numerous neurites to their corresponding cell body. However, it can be seen that a number of neurites pass over the pixel. Signals from these neurites will mix indistinguishably when recorded at the low spatial resolution of the high speed camera. Scale bar in **C** applies to all panels.

**Figure 2:**
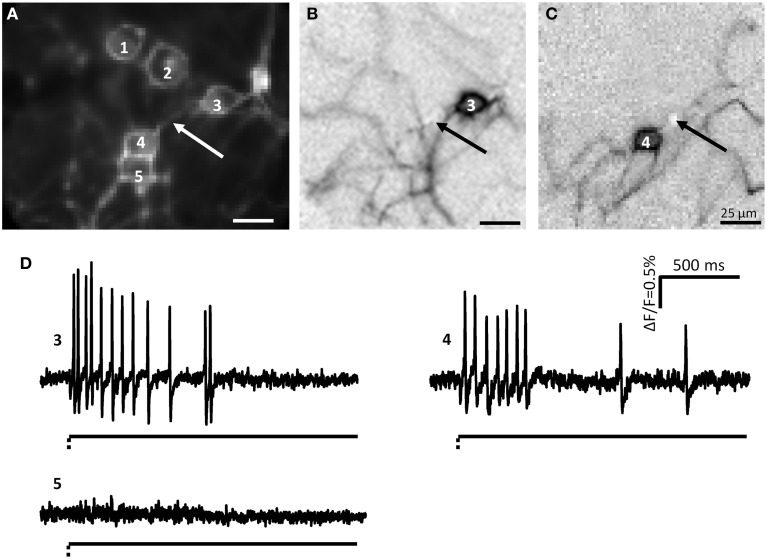
**Myenteric neurons fired action potentials after deformation of their neurites by von Frey hair stimulation. (A)** Shows a picture taken by the CCD camera of a cluster consisting of 5 neurons. The white arrow marks the site of mechanical deformation. Neurons 3 and 4 responded to deformation with spike discharge. In **(B,C)** the independent component analysis (ICA) images of the mechanosensitive neurons 3 **(B)** and 4 **(C)** are shown. The spot of carbon fiber stimulation is marked by black arrows. The traces of the responses of neurons 3, 4, and 5 are shown in **(D)** Neurons 3 and 4 are mechanosensitive, neuron 5 not. The broken and solid lines beneath the traces illustrate the fast dynamic and sustained deformation.

In addition to the tools provided by the Neuroplex software (RedShirt Imaging) we used the “ICA.” The fast ICA algorithm (Hyvärinen, 1999) was employed using programs in Igor in Igor Pro 6.22A (Wavemetrics Inc., Lake Oswego, OR, USA) and R (R Core Team, 2013) to reconstruct images of individual neurons. The ICA is a method to separate multivariate signals into additive subcomponents (“sources”) and has been already applied to neuroimaging data (Brown et al., 2001). Briefly, the complete dataset was first filtered with a bandpass filter (3–300 Hz). Next, the filtered data was preprocessed by a Principal Component Analysis (PCA) with 20 or 40 components, depending on the approximate number of neurons in the field of view. As the PCA for datasets with this size is very time consuming, we used the implicitly restarted Lanczos bidiagonalization algorithm (IRLBA, package irlba 1.0.3 in R) (Baglama and Reichel, 2005) to compute a partial singular value decomposition of the data to compute only the first 20 or 40 PCA components. We verified that this approach yielded the same results as a “full” PCA. However, while the computation with the full PCA took about 30 min, the use of the IRLBA algorithm reduced this to less than 2 min (using a computer with an Intel i3 processor with 2.27 GHz, 3 GB RAM and Microsoft Windows 7, 32 bit). The PCA components were then further processed by the fast ICA algorithm in Igor. This finally produced the “source” signals and images that showed the contribution of each individual pixel to these sources. This approach was chosen for three reasons. First, to separate signals from individual neurons that happen to occur on 1 pixel. Second, to follow signals from the soma to all neurites. This is not possible by manually choosing individual pixels because of the unfavorable signal to noise ratio in signals from single pixels. Third, to filter out signal artifacts due to mechanical deformation, because the ICA separates artifacts and neural signals into different sources. The ability of the ICA to separate signals on single pixels is illustrated in Figure 1. The image obtained with the ICA reveals the soma with its corresponding neurites that conduct the electrical signal. The signals can be then seen at the site of mechanical stimulation along neurites, in the soma and into other neurites (Figure 2). Discharge patterns of action potentials were always confirmed in the original data traces. We never found an example of a discharge pattern in the ICA that we could not reproduce in the original traces. It needs to be emphasized that original traces were used to analyse signals with a time delay, e.g. propagation of spikes along neurites and invasion of spikes into the soma or neurites.

The traces obtained with the ICA were further analyzed with the Macro “Neuromatic” in Igor Pro (Think Random, London, UK). For every trace a threshold for counting peaks was individually set. The peaks that were counted as action potentials were further analyzed with an enlargement tool by visual controlling for clear action potential shape. For calculation of the burst spike frequency the number of action potentials was divided by the time from the first to the last action potential fired.

In order to study the adaptation pattern of MEN we adopted an adaptation index (AI) comparing the firing frequency between the first 500 ms and the remaining 1500 ms independent from the method used to evoke mechanical stimuli: von Frey hair in the present study or intraganglionic injection in our previous study in intact tissue (Mazzuoli and Schemann, 2009).

The AI was calculated with the following equation:

$$\text{AI}=\frac{\text{action potential frequency }500-2000\text{ms}}{\text{action potential frequency }0-500\text{ms}}.$$

This AI calculation was used to differentiate rapidly from slowly or ultra-slowly adapting firing behavior. Neurons having an AI = 0 were defined RAMEN, neurons with AI between 0 and 1 were defined SAMEN and neurons with an AI ≥ 1 USAMEN.

Neuronal deformations were analyzed by spotting movement artifacts in the original traces. In addition we used the “Scan Movie” tool in the Neuroplex software to identify the deformed regions.

All data are presented as median with their 25 and 75% quartiles. Differences are considered as significant when *p* < 0.05.

To identify different neurites of the same neuron we used the image obtained by the ICA in response to nicotine application. We assumed that probing of even large fiber tract deformed all neurites because the carbon fiber was at least the diameter of the fiber tract. We analyzed the number of mechanosensitive neurites from each neuron and we compared the spike frequency of 3 mechanosensitive neurites belonging to the same neuron with a Friedman Repeated Measures Analysis of Variance (ANOVA) on Ranks. To statistically analyze the differences in the RAMEN/SAMEN/USAMEN distributions the analysis of residual method (Haberman, 1973) was implied.

The conduction velocity was measured in recordings with 10 kHz sampling frequency. With this frame rate the field of view of the CCD camera was reduced from 80 × 80 pixels to 80 × 12 pixels. We measured the delay between two signals at most distant regions in the field of view.

## Results

### Mechanical stimulation of myenteric neurites evoked action potentials

**Figure 3:**
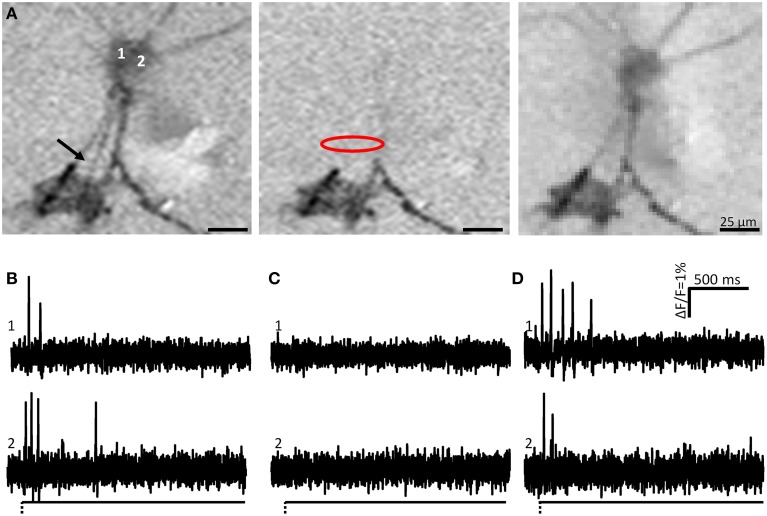
**Soma spike appearance after neurite deformation is not caused by pulling the soma, i.e., the site of mechanical stimulation is the site of the spike generation. (A)** Shows the ICA images of **two** neurons (1 and 2) after neurite stimulation indicated by the black arrow. The image to the left is the control response (soma spikes shown in **B**). Somata of both neurons were activated after neurite deformation. Central image represents activity during local application of tetrodotoxin (TTX) (soma spikes shown in **C**). Site of TTX application is indicated by the red oval. Note that spike propagation toward the soma is blocked and only occurs at neurite regions distal to the TTX application site. The image to the right is after wash out of TTX (soma spikes shown in **D**). Note that soma spikes recovered.

During culture, enteric neurons formed a ganglionated network with neuronal clusters interconnected by fiber tracts consisting of several neurites. Therefore, probing these fiber tracts might have deformed more than one neurite and hence we frequently observed activation of more than one neuron in the field of view (Figure 2). However, applying ICA to discriminate between different spike discharge patterns we were able to distinguish the responses of somata and neurites between different neurons (Figures 1, 2). We probed 256 neurites in 203 clusters (30 guinea pigs) and evoked spike discharge in 45% (114 out of 256) of the probed neurons. Action potentials were detected in the probed neurite, propagated toward the corresponding soma and frequently invaded neurites that were not mechanically stimulated (Figure 2). Neurons fired at a burst spike frequency of 13.4 (5.9/20.1) Hz for a period of 1080 (418/1576) ms. The distance from the site of mechanical stimulation on the neurite to the edge of the soma eliciting a sensory response ranged from 4 and 155 μm. We wanted to rule out that soma spikes after neurite probing were caused by pulling on the soma while deforming the neurite. We never observed this, but minimal deformations not evident with our spatial resolution may still occur. In order to demonstrate that the action potentials indeed originated in the neurite at the site of deformation, we locally applied TTX via a small-tipped pipette just only over a region between the mechanical stimulation and the soma. We validated our method of local blockade of spike conduction by showing that the local TTX application blocked the soma invasion of action potentials induced by electrical stimulation (21 clusters, 2 guinea pigs). Local TTX application prevented the soma spikes invasion which recovered 10 min later after TTX has been washed away by the continuous tissue perfusion (Figure 3). We therefore conclude that soma spike invasion was indeed caused by neurite probing.

To calculate the conduction velocity we followed spike propagation along neurites of 5 neurons. The calculated conduction velocity was 0.19 ± 0.06 m/s. This is similar to values previously reported in the ENS (Nishi and North, 1973; Hendriks et al., 1990; Schemann et al., 2002).

### Mechanosensitivity of neurites is not restricted to single sites (hot spots)

To test if enteric neurons have many mechanosensitive sites on their neurites we stimulated 13 mechanosensitive neurites in 5 clusters (4 guinea pigs) at 3 different locations. The first stimulation was farthest from the soma. 10 neurons (77%) showed a spike discharge in response to all 3 stimulations whereas 3 neurons were activated only by two of them. This suggested that a large part of the neurites were mechanosensitive as opposed to specialized mechanosensitive hot spots only expressed at their endings. The latter was a characteristic feature of extrinsic visceral afferents (Zagorodnyuk and Brookes, 2000; Zagorodnyuk et al., 2001). In addition, probing at all three sites evoked similar burst spike frequencies: 10.0 (3.4/21.3) Hz vs. 7.8 (3.2/15.4) Hz vs. 6.1 (4.8/11.8) Hz.

### Morphology of mechanosensitive myenteric neurons

We used the ICA image to morphometrically characterize 151 mechanosensitive neurons by measuring the large and small diameter and the number of neurites. The large diameter was 20 (16/22) μm (range: 9–46 μm) and the small diameter was 14 (12/16) μm (range: 5–24 μm). The majority showed a multipolar morphology, having an average 3 (2/4) (range: 1–7) neurites.

**Figure 4:**
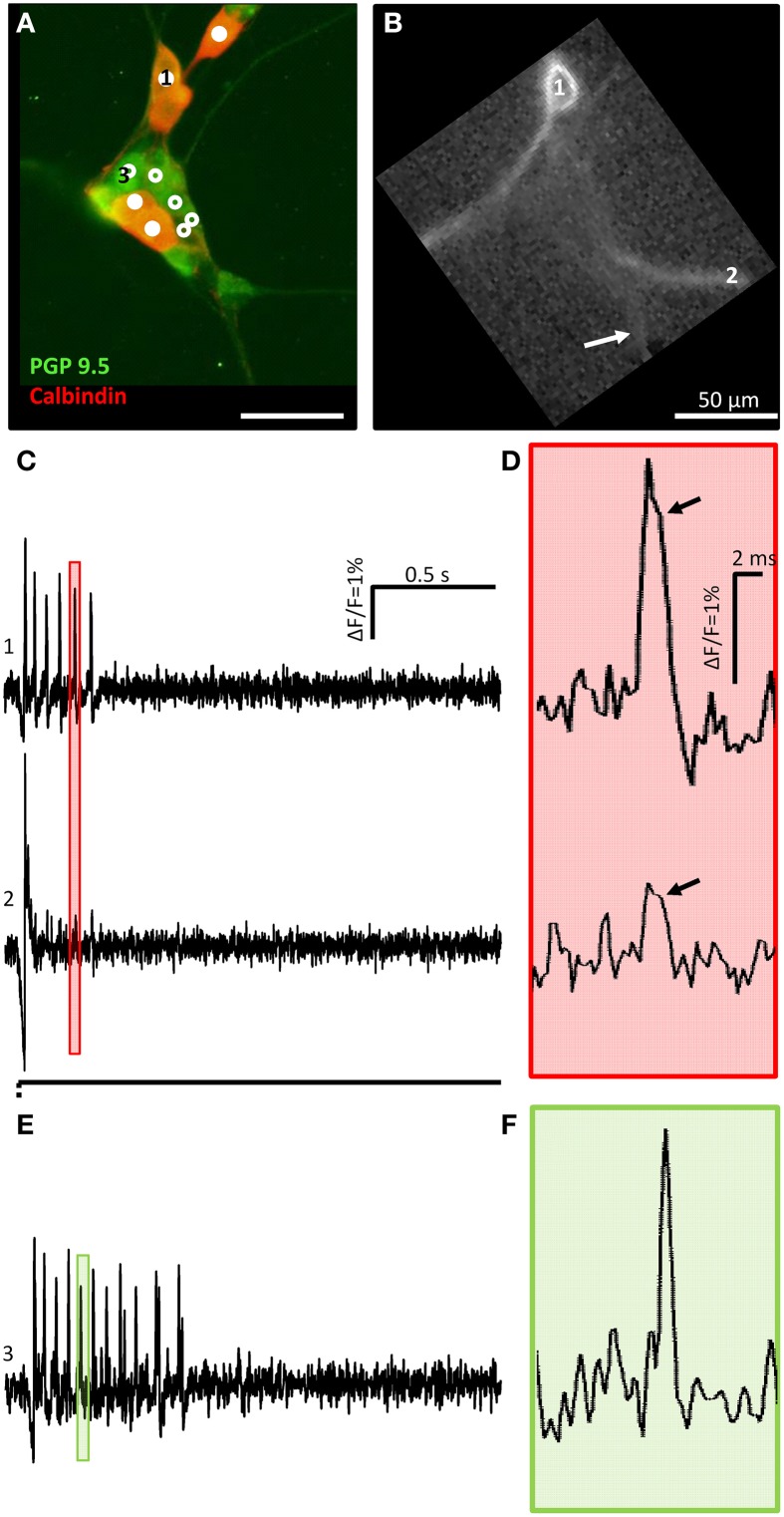
**A calbindin positive mechanosensitive enteric neuron (MEN) fires action potentials with a hump during down stroke of soma and neurite spikes. (A)** Shows a cluster with 10 MEN activated by neurite deformation indicated by an arrow in **(B)**. PGP 9.5 immunoreactivity in green was used as a pan neuronal marker. Calbindin positive (mixed red and green) or negative (only green) MEN are marked by white dots or white circles, respectively. The ICA image of one calbindin positive MEN is shown in **(B)**. The traces in **(C)** show the soma (marked by 1 in **A,B**) and neurite (marked by 2 in **B,C**) responses to mechanical deformation (white arrow) of a neurite. In **(D)** the pink shaded area marked in **(C)** is shown as an expanded trace. The soma as well as the neurite spike showed a hump during spike down stroke marked by black arrows. **(E)** shows the trace of the calbindin negative MEN marked with 3 in **(A)**. In **(F)** the green shaded area marked in **(E)** is zoomed in.

We used double immunofluorescent labeling with the pan neuronal marker PGP 9.5 and calbindin to specifically address the question whether mechanosensitive neurons correspond to the previously identified IPANs (Li and Furness, 1998; Quinson et al., 2001). Only 16 out of 72 mechanosensitive neurons were calbindin positive (22%) (Figure 4A). Noteworthy calbindin mechanosensitive neurons had a large diameter of 14 (12/15) μm (range 11–16 μm) and a small diameter of 11 (10/13) μm (range 9–13 μm). All mechanosensitive calbindin positive neurons showed a Ca^2+^ hump on the falling phase of their spikes (Figure 4D) which suggested that they indeed were AH neurons which fired Ca^2+^ spikes (Clerc et al., 1998). The hump was detectable on the soma and on the neurites (Figures 4C,D) and is not present on the spikes of calbindin negative neurons (Figures 4E,F). We measured the spike duration of 13 calbindin positive neurons vs. 16 non-calbindin neurons and we found a statistical significant difference: 3.7 ± 0.8 ms vs. 3.0 ± 1.0 ms (*p* = 0.04).

### Responses of multipolar neurons to multifocal mechanical stimulation

**Figure 5:**
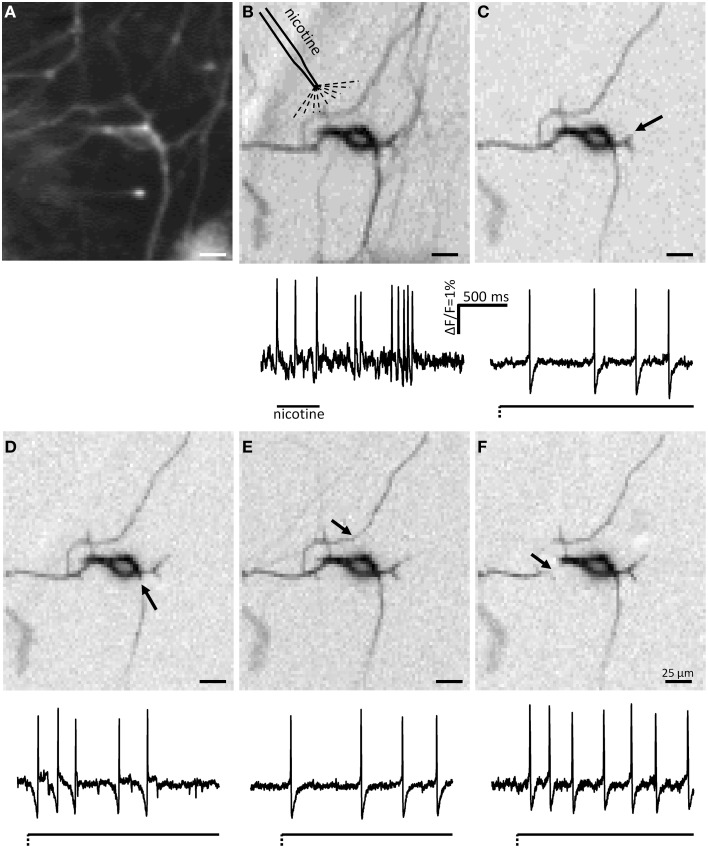
**ICA images and spike discharge patterns of a mechanosensitive neuron in response to nicotine and to deformation of several neurites**. The CCD camera picture of a neuron is shown in **(A)**. The ICA image of the nicotine response is shown in **(B)**. **(C–F)** Show the ICA images of the responses to mechanical deformation of different neurites of the same neuron. The sites of deformations are marked by black arrows. Very few neurites activated by nicotine application **(B)** are not detectable after deformation of different neurites **(C–F)**. It is evident that mechanosensitive neurites are also invaded by soma spikes generated by deformation of other neurites. The firing behavior of the neuron was slowly adapting independent of which neurite was deformed.

The ICA images clearly revealed that mechanosensitive myenteric neurons had several signal conducting neurites (Figure 5). To study if all neurites of an individual neuron were mechanosensitive we probed different neurites belonging to the same soma with the carbon fiber. Prior to probing we identified the neurites belonging to a particular soma with the ICA image generated by spike discharge in response to nicotine (Figure 5). The rationale behind this experimental approach was that nicotine would evoke dendrites/somal spikes which invaded other neurites (likely axons). We observed that the ICA image generated after mechanical stimulation closely resembled, but was not 100% identical, the one obtained in response to nicotine (Figure 5).

**Figure 6:**
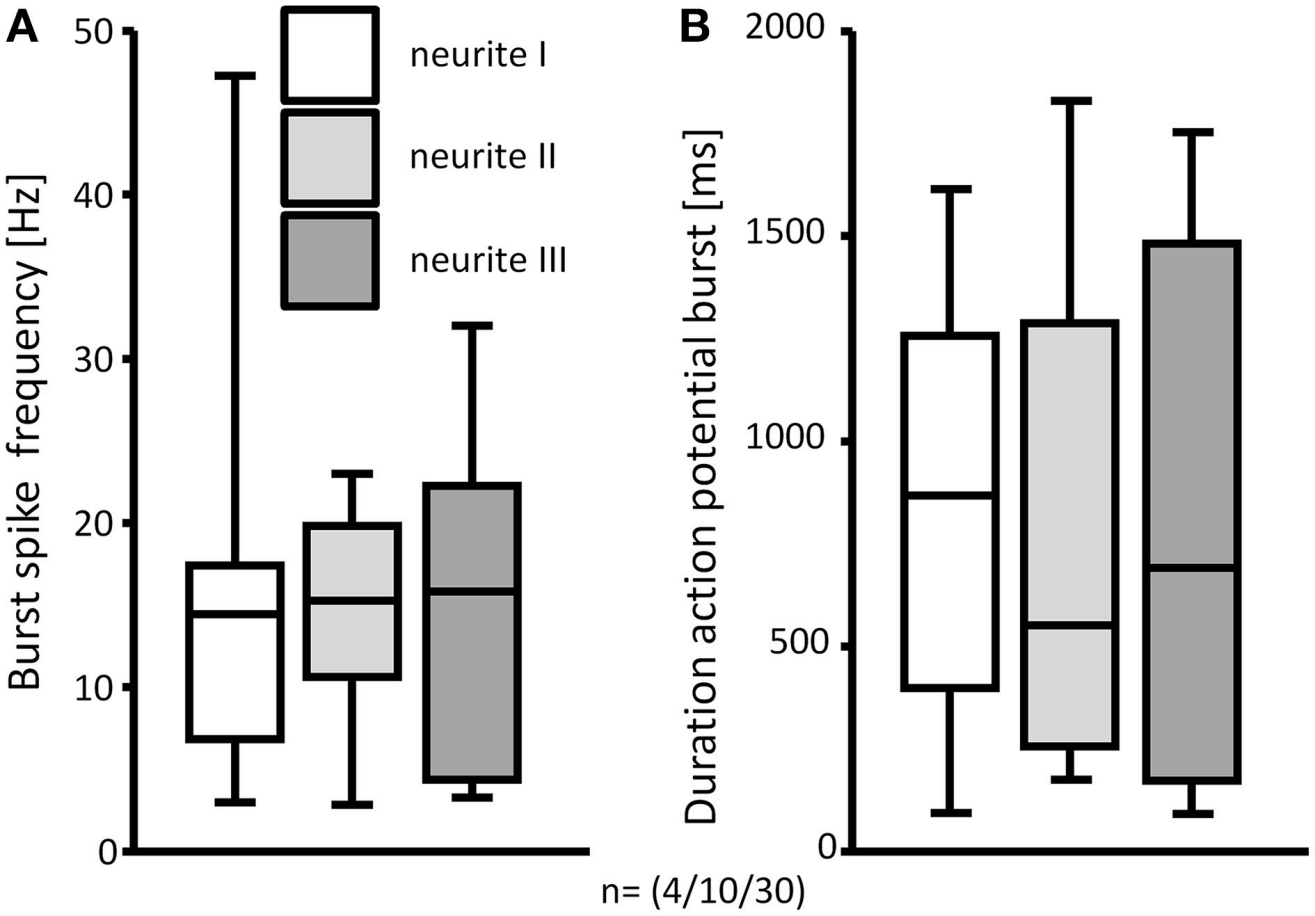
**The response to deformation of 3 mechanosensitive neurites of the same neuron was not different**. Burst spike frequencies **(A)** and durations of the spike bursts were comparable **(B)**. N numbers (guinea pigs/clusters/neurons) are shown in brackets.

All in all, the soma and neurites of 52 neurons from 15 clusters derived from 4 guinea pigs were analyzed. We classified these neurons by the number of mechanosensitive neurites. Eight of these neurons were not mechanosensitive at all. In 21 neurons all probed neurites responded to carbon fiber stimulation. In contrast, 24 neurons exhibited at least 1 neurite which did not respond to mechanical stimulation: in 3 neurons 3 of 4 probed neurites were mechanosensitive, in 4 neurons 2 of 3 probed neurites, in 2 neurons 2 of 4 probed neurites, in 6 neurons 1 of 2 probed neurites, in 4 neurons 1 of 3 probed neurites, in 2 neurons 1 of 4 probe neurites and in 2 neurons 1 of 5 probed neurites. In those neurons that had at least 3 mechanosensitive neurites we compared the spike discharge patterns in response to probing each neurite. Probing different neurites from the same neuron evoked similar responses (Figure 6). Probing different neurites of the same neuron revealed that most neurites serve afferent and efferent functions (Figure 5). This was the conclusion from the finding that a mechanosensitive neurite was invaded by spikes when another neurite was deformed (Figure 5).

### Mechanosensitivity of the soma

**Figure 7:**
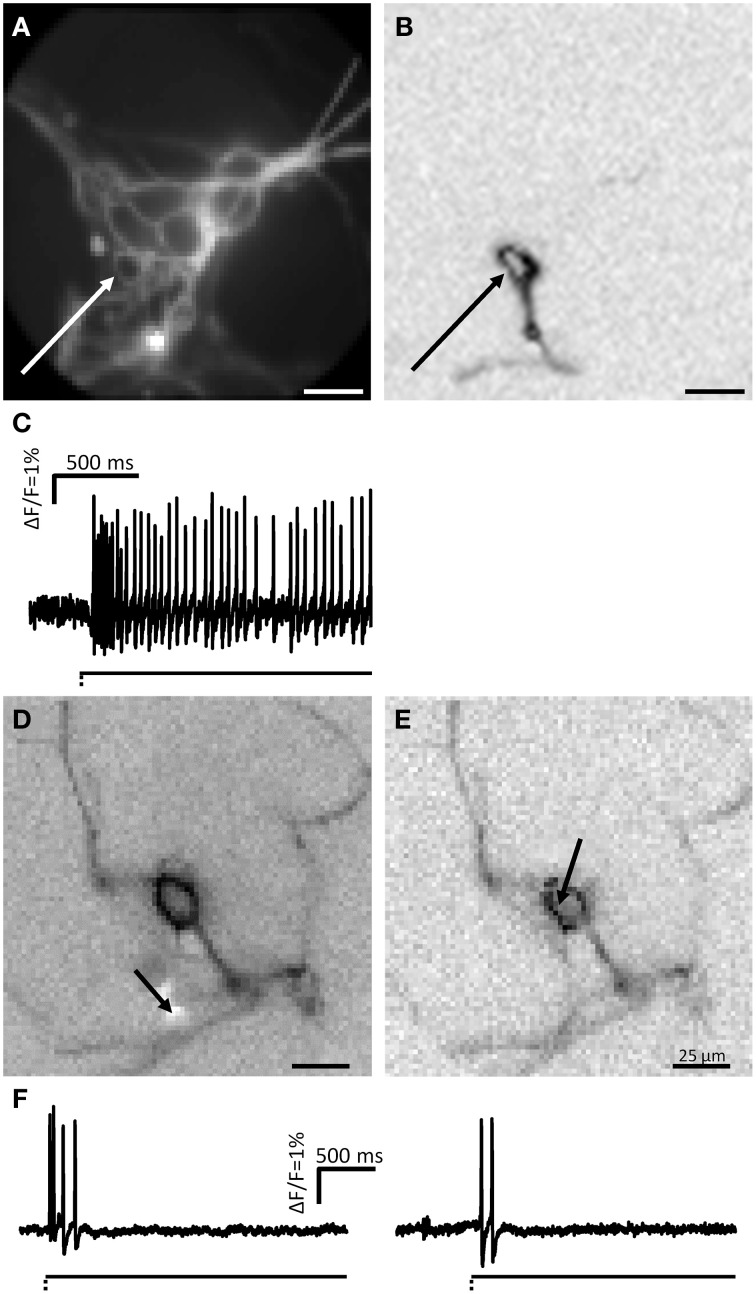
**MEN fire action potentials after soma deformation. (A)** Shows a picture taken by the CCD camera of a cluster. The soma of one neuron was deformed at the spot marked by a white arrow. In **(B)** the ICA image of the deformed neuron is displayed. **(C)** Shows the spike discharge pattern of this neuron. Paired stimulation (soma and neurite of the same neuron) showed that electrical signal was conducted in the same number of processes. **(D,E)** Show the ICA images of one neurons stimulated on a neurite **(D)** and on the soma **(E)** revealed the same number of neurites. **(F)** Shows the spike discharge corresponding to the neurite and soma stimulations, respectively.

We used the carbon fiber to stimulate the somata of 96 neurons in 67 clusters (18 guinea pigs). Targeted soma deformation induced spike discharge in 13 of 96 neurons (14%). This is significantly less than the proportion activated by neurite probing. The action potentials were detectable on the stimulated soma and invaded also neurites (Figures 7A–C). The spike burst features were similar to those evoked by neurite stimulation (Table 1). Paired stimulations (soma and neurite) were performed in 18 mechanosensitive neurons. The somata of neurons which responded to neurite stimulation were mechanically stimulated. Only 45% of the neurons responding to neurite deformation responded also to soma deformation. Responses to soma deformation after stimulation of the neurite were similar to those obtained during soma deformation without prior neurite stimulation [burst frequency 17.9 (9.4/26.4) Hz vs. 16.9 (7.4/23.7) Hz]. The neurons stimulated in a paired manner showed in the ICA images the same number of activated neurites (Figures 7D–F).

**Table 1:** **Firing behavior of mechanosensitive neurons in the guinea pig (GP) myenteric plexus (MP) after neurite or soma deformation, in GP dorsal root ganglia (DRG) after neurite deformation and in human MP after neurite deformation**.

	**GP MP soma deformation**	**GP MP neurite deformation**	**Human MP neurite deformation**	**GP DRG neurite deformation**
Burst spike frequency [Hz]	16.9 (7.4/23.7)	13.4 (5.9/20.1)	6.0 (4.1/11.1)^*^	8.9 (6.7/16.7)
Duration spike burst [ms]	1338 (875/1680)	1080 (418/1576)	1414 (1077/1669)^**^	1503 (760/1739)^**^
Adaptation Index	0.3 (0.0/0.6)	0.2 (0.0/0.5)	0.4 (0.3/1)^*^	0.3 (0.1/0.5)
Firing behavior:		^***^	^***^	^***^
Rapidly adapting	44	28	6	16
Slowly adapting	50	63	65	74
Ultra-slowly adapting [%]	6	9	29	10
*N* = (GP or human tissue/ cluster / neuron)	(8/14/18)	(25/87/146)	(10/35/85)	(2/22/63)

* *marks significant differences in the burst spike frequency and in the AI tested with ANOVA and Dunn's Method (p ≤ 0.001) between GP and human*.

** *marks significant differences in the duration of the spike burst between DRG and GP neurite and between human and GP neurite deformations tested with a One-Way ANOVA and Dunn's Method (p ≤ 0.001)*.

*** *marks significant difference in the adjusted residuals of the neuronal distributions tested with analysis of residual method (Haberman, 1973)*.

### Spike discharge pattern of men after neurite and soma deformation

**Figure 8:**
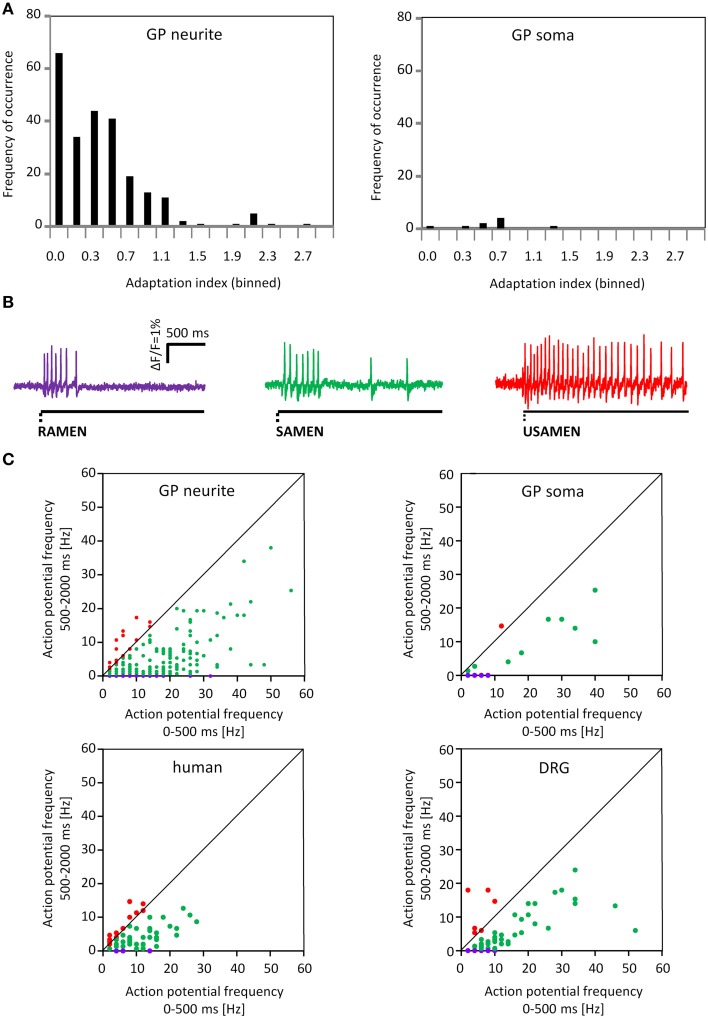
**Adaption indices indicate rapidly, slowly and ultra-slowly adapting firing behavior. (A)** Shows the distribution of the adaptation index (AI) for the neurons after neurite or soma deformation. On the x axis the AI is represented while on the y axis the frequency of occurrence is showed. **(B)** Shows 3 representative traces for Rapidly, Slowly and Ultra Slowly Adapting MEN; RAMEN, SAMEN and USAMEN, respectively. **(C)** Shows the spike frequency during the first 500 ms plotted against the spike frequency during the last 1500 ms. The 2 upper graphs represent data for guinea pig MEN after neurite and soma deformation. The two lower graphs illustrate data from human MEN and guinea pig dorsal root ganglia (DRG) neurons after neurite stimulation. The firing behavior is color coded: purple represent rapidly, green slowly and red for ultra-slowly adapting neurons.

Firing of MEN showed different adaptation behaviors. The AI was calculated for 257 mechanosensitive neurons of which 239 were stimulated on the processes and 18 on the soma. The AI for the neurons responding to the process stimulation was 0.2 (0.0/0.5) while the AI for the ones stimulated on the soma was 0.3 (0.0/0.6). Based on the AI we could define three neuronal populations (Figure 8). The first one was represented by nerve cells that have an AI of 0 meaning that these cells responded only during the first 500 ms and therefore behaved as RAMEN. After process and soma stimulation we encountered 28% (66 of 239) and 44% (8 of 18) RAMEN, respectively. The second one was made of neurons with an AI between 0 and 1: these neurons referred to as SAMEN fired also during the second recording period (500–2000 ms) but with a gradually decreasing frequency. We identified 63% (152 of 239) and 50% (9 of 18) SAMEN after process and soma stimulation, respectively. The third population comprised those neurons with an *AI* ≥ 1. These made up 9% (21 of 239) and 6% (1 of 18) after neurite and soma stimulation, respectively. These neurons obviously did not adapt during the recording time. We assume that also these responses will eventually adapt because we found that their percentage decreased from 9 to 5% (6 of 124) when we prolonged the recording period to 10 s. We therefore referred to these neurons as ultra-slowly adapting mechanosensitive enteric neurons (USAMEN). In few experiments we left the von Frey hair in place for several minutes and recorded the responses after 1 or 2 min. In no case we did observe spike discharge during sustained compression suggesting that there was eventually adaptation. The proportion of RAMEN, SAMEN and USAMEN were significantly different between neurite and soma stimulation (Table 1).

Noteworthy all calbindin immunoreactive neurons were RAMEN (Figure 4).

**Figure 9:**
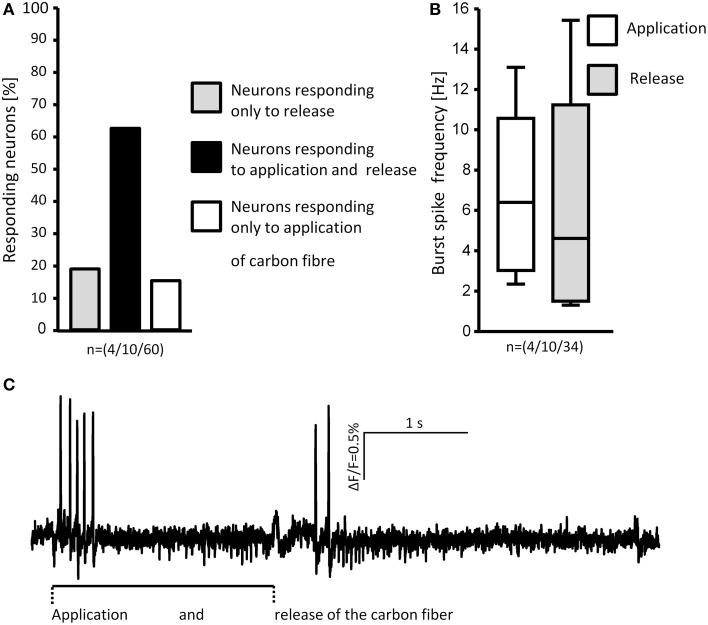
**Loading and unloading (retracting) the von Frey hair evoked responses in MEN**. The percentages of neurons responding to either one or both stimuli are shown in **(A)**. Comparison of the burst spike frequencies of neurons responding to both stimuli shows no significant difference **(B)**. The spike discharge of a representative neuron in response to application and release of the stimulus is shown in **(C)**. N numbers (guinea pigs/clusters/neurons) are shown in brackets.

The rapid adaptation was not due to desensitization to deformation because unloading, i.e., retraction of the von Frey hair evoked again spike discharge (Figure 9). Spike burst frequencies during onset and release of deformation were similar.

### Primary culture of human myenteric neurons show similar behavior in response to mechanical stimulation

We cultured human myenteric neurons from surgical specimens in order to obtain first data on translational aspects. Experiments were performed on 64 clusters from 19 human tissue samples. Pressing the carbon fiber on neurites caused spike discharge in 34% of all the probed neurons (86 out of 251). In principle, the proportion of responding neurons and pattern of responses were similar to those observed in cultured guinea pig myenteric neurons, although the burst spike frequency, the burst duration, the adaptation index were significantly different (Table 1, Figure 8C). Response of neurons isolated from small and large intestinal samples were not different: burst spike frequency was 6.0 (4.3/11.4) vs. 6.0 (2.7/11); the duration of the spike burst was 1343 (1088/1632) vs. 1737 (0581/2044), the AI was 0.4 (0.2/1) vs. 0.4 (0.3/0.8) and the proportion of RAMEN, SAMEN, and USAMEN was 6/72/22 vs. 14/64/22.

### Response of DRG neurons to deformation

In a proof of principle study we tested in cultured DRG neurons the validity of our carbon fiber stimulation protocol. Moreover, this allowed us to compare electrophysiological characteristics of MEN with those of well-characterized mechanosensors in the central nervous system.

**Figure 10:**
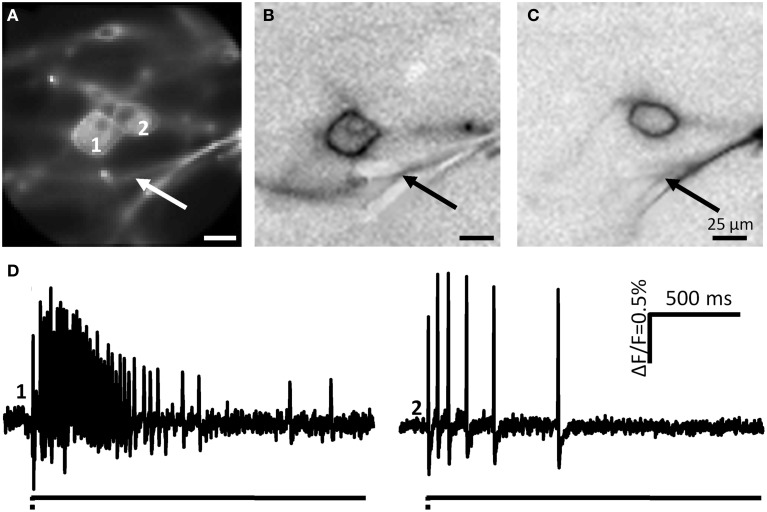
**Neurite deformation activates cultured DRG neurons. (A)** Shows a picture of two DRG neurons deformed at the spot indicated by the white arrow. **(B,C)** Represent the ICA images of the two neurons. Site of deformation is marked by black arrows. In **(D)** the spike patterns of the two mechanosensitive neurons are displayed.

We stimulated neurites of 54 DRG neurons (2 guinea pigs) with the carbon fiber and evoked a response in 65% of them (Figure 10). In principle, the spike discharge patterns were quite similar between mechanosensitive DRG and enteric neurons with two noteworthy exceptions (Table 1). Firstly, the spike burst duration was significantly longer in DRG neurons. Secondly and most strikingly, soma deformation of 21 DRG neurons never evoked spike discharge. The AI for the mechanosensitive DRG neurons was 0.3 (0.1/0.5) with 16% (10 of 61) RAMEN, 74% (45 of 61) SAMEN, and 10% (6 of 61) USAMEN (Figure 8C).

## Discussion

With this study we report spike discharge behavior of primary cultured mechanosensitive myenteric neurons after targeted deformation of their neurites or soma. Applying ICA allowed us to reconstruct the morphology and the signaling of MEN. We thereby demonstrated some new and unique properties of mechanosensitive myenteric neurons. The main findings were: firstly, mechanosensitivity occurred along enteric neurites and was not restricted to hot spots at terminal endings. Secondly, once initiated in the neurites, the spikes propagated toward the soma and invaded other neurites. Thirdly, neurites had afferent as well as efferent functions. Fourthly, spike invasion in neurites was controlled by the excitability state of the soma. Fifthly, neurites were more sensitive to deformation than the soma. Also, there were rapidly and slowly adapting mechanosensors; the rapidly adapting ones were more often encountered after soma deformation. Finally, we showed that cultured human myenteric neurons were also mechanosensitive with similar properties to guinea pig myenteric neurons.

We found that around 50% of the primary cultured myenteric neurons possessed mechanosensitive properties. The duration of their spike burst lasted several hundred milliseconds, which is well in the range of the duration of muscle contractions (Schemann et al., 1985). Mechanosensitive properties were not only confined to a single neurite but rather present on several processes of one neuron. In our study, neurite as well as soma deformation caused spike discharge. Distortion of neurites has been shown to evoke somal spikes in freshly dissected guinea pig myenteric plexus preparations (Kunze et al., 2000). However, in the same study, where only AH neurons were probed, spike discharge in mechanosensitive neurons was inhibited during soma compression through the patch pipette (Kunze et al., 2000). There are several possible explanations for the contradictory findings. It seems possible that the rather “invasive” recording techniques with patch clamp or sharp electrode affected responses in mechanosensitive neurons. As reported previously, patching cells can change their mechanical properties due to disruption of normal membrane structure and redistribution of ion channels at the site of the patch (Suchyna et al., 2009). In addition, patch clamp and sharp electrodes differentially affect features of the slow AH, which is a hallmark of IPANs (Mao et al., 2006). Patch clamp studies of IPANs have been performed after the ganglion has been exposed to proteases, which excited enteric neurons after brief application but desensitized them when applied for longer time periods (Mueller et al., 2011). It is unlikely that the different responses of mechanosensitive myenteric neurons to soma deformation were due to different electrophysiological behavior of IPANs vs. MEN. This notion was supported by our finding that spike discharge was also evoked after soma stimulation of calbindin expressing AH neurons, both features of IPANs in the guinea pig ileum myenteric plexus (Quinson et al., 2001). As also discussed earlier, some of the previously described multifunctional RAMEN or SAMEN share common properties with IPANs (Mazzuoli and Schemann, 2009; Schemann and Mazzuoli, 2010).

In order to not only describe but also distinguish mechanosensitive neurons by their spiking pattern we calculated an adaptation index that allowed us to classify them in three categories based on the firing adaptation. We found rapidly, slowly and ultra-slowly adapting neurons. The majority of the mechanosensitive neurons in culture appeared to be slowly adapting, both after neurite and soma deformation. In contrast, firing of most mechanosensitive neurons in intact, non-dissociated guinea pig myenteric plexus adapted rapidly (Mazzuoli and Schemann, 2009). However, in this study we did not calculate the AI. In order to better compare the results of our two studies we reanalyzed the data from the previous study and calculated the AI in the same way. The AI was significantly lower than in cultured myenteric neurons [0.0 (0.0/0.2; *p* < 0.001; Mann–Whitney Rank-Sum Test)] because the proportion of RAMEN was much higher; 60% (83 of 138) of the mechanosensitive neurons were RAMEN, 36% (49 of 138) were SAMEN and 4% (6 of 138) were USAMEN. The differences could of course be simply caused by plasticity induced during culturing. As a more likely explanation we suggest differences in stimulus modalities although a final explanation for the differences requires a dedicated study. Different stimuli were probably the reason for the broad range of firing patterns of mechanosensitive myenteric neurons reported in the literature (Mayer and Wood, 1975; Kunze et al., 1998, 2000; Spencer and Smith, 2004; Mao et al., 2006; Mazzuoli and Schemann, 2009. In our previous studies intraganglionic volume injection deformed the ganglion neurons in a dynamic fashion for 400 ms followed by a sustained deformation for the rest of the recording periods of several seconds. This mechanical stimulus was a mixture of compression, elongation and shear stress. In the present study we used brief compression with a von Frey hair. In addition, the two studies differed with regard to the site of stimulation. While the present study allowed targeted deformation of the neurites or the soma, the intraganglionic injection might have primarily deformed the somata. It is noteworthy, that firing in cultured myenteric neurons adapted faster after soma compared to neurite deformation.

Thus the most striking and at the same time puzzling finding was that mechanosensitive neurites not only generated spikes that propagate toward the soma but also conduct spikes generated in other neurites or the soma. This conclusion was based on the finding that neurite deformation caused invasion of spikes into other neurites, which also respond to deformation. The ICA image of neurons responding to deformation clearly showed neurites conducting in both directions. Moreover, nicotine application caused spike discharge in the soma and along neurites, including those that were mechanosensitive. At this point, we have no conclusive explanation how myenteric neurons prevent collision of action potentials once several mechanosensitive neurites are activated. Since the ICA images of the nicotine showed in a few cases more neurites compared to the image of the mechanical responses, we could speculate that some kind of gating process is occurring into the soma.

Our results suggested that most neurites of mechanosensitive myenteric neurons were activated by deformation. We cannot exclude that this was a phenomenon of plasticity in cultured neurons which might result in excess expression of mechanosensitive channels. Evidences for altered features in cultured sensory neurons have been described in DRG neurons (Delrée et al., 1993) although they retained their main properties as nociceptors (Gold et al., 1996). The ICA revealed the morphology of each mechanosensitive neuron based on independent spike discharge patterns. Whereas cultured DRG neurons are all well-characterized pseudounipolar neurons (Dogiel, 1908; Kandel et al., 2000) myenteric neurons have different morphologies and are classified as Dogiel type I, Dogiel type II, and Dogiel type III neurons (Dogiel, 1899). It was not possible in our study to unequivocally classify each neuron according to these categories. Most of them had multiple action potential conducting neurites, but differed in size. Although we have not observed a particular spatial organization of these mechanosensitive neurites, there may be a preferred projection and a spatial preference of mechanosensitive neurites during development in the gut wall, which assures polarized activation of the soma from only one direction.

The finding that neurites conducted spikes orthodromically and antidromically suggested that they could have afferent as well as efferent or integrative functions. In principle this conclusion agrees with our previous findings that mechanosensitive myenteric neurons in intact tissue were multifunctional (Mazzuoli and Schemann, 2009). This concept was based on the result that deformation of myenteric ganglia in guinea pig ileum directly activated neurons generally considered as motor- or interneurons (Mazzuoli and Schemann, 2009) and was further supported by the identification of sensory interneurons in the myenteric plexus of the guinea pig colon (Spencer and Smith, 2004). Nevertheless, this finding does not make enteric neurons exotic examples of sensory neurons as recent studies suggested a shift in paradigm in that the strict classification of neurites by a sole function must be questioned. For example, it has been shown that axons can perform diverse functions, such as modulation of spike shape or integration of somatic spikes that extend beyond the prevailing model of axon physiology (Sasaki, 2013).

It needs to be emphasized that we validated our recording and stimulation techniques with studies on DRG neurons, which peripheral nerve endings represent well-established mechanosensors transmitting the information from the periphery to the central nervous system (Hogan, 2010). Using the same mechanical stimulus paradigm we observed some common but also different features of mechanosensitive myenteric and mechanosensitive DRG neurons. The discharge pattern in response to neurite stimulation was similar although the spike burst lasted much longer in DRG neurons. We observed spike discharge in DRG neurons only after neurite distortion but never after soma deformation. This might reflect the anatomical situation in that the cell bodies of DRG neurons are *in vivo* protected by the backbone and are therefore not directly exposed to mechanical stress. Activation of the soma of DRG neurons required much stronger stimuli than those necessary to activate neurites (Hu and Lewin, 2006). We therefore conclude that the stimulus strength required to activate DRG or myenteric neurites was similar whereas the soma of myenteric neurons is much more sensitive to deformation.

Only 45% of those myenteric neurons that responded to neurite distortion responded to soma deformation. This suggested that neurites possessed a higher sensitivity to deformation than the soma. It is unknown which forces act on neurons during muscle contraction and relaxation. In the present study we used stimulation strength of 0.4 mN. One may argue that mechanical stimulation restricted to such a small area may never appear under physiological conditions. This is true, but we used in this study the targeted stimulation as a tool to reveal the properties of the mechanosensitive neurons. The response to carbon fiber stimulation appeared to represent a genuine, physiologically relevant response. The stimulus strength used in our study is in the range of that used by others. We previously showed that von Frey hairs exerting forces above 1.0 mN activated ileal myenteric neurons in intact tissue (Mazzuoli and Schemann, 2009). Mechanoreceptors of rectal intraganglionic laminar endings (IGLEs) were activated by forces between 0.08 and 7 mN (Lynn et al., 2003), IGLEs in the stomach responded to a 0.4 mN von Frey hair (Zagorodnyuk et al., 2001) and enteric viscerofugal neurons fired action potentials when probed with 0.8–5 mN von Frey hairs (Hibberd et al., 2012).

Local deformation at multiple locations along the neurites evoked action potentials. Similar findings were observed for cultured DRG neurons (Usoskin et al., 2010). In the gastrointestinal tract the terminal endings of extrinsic visceral afferent fibers have been shown to express specialized hotspots as the site of mechanotransduction (Zagorodnyuk and Brookes, 2000; Zagorodnyuk et al., 2001; Lynn et al., 2003). Our findings suggested that mechanosensitive myenteric neurites have no specialized hot spots but mechanosensitivity appeared to be a feature of the entire neurite, which allows myenteric neurons to encode mechanical stimuli at different sites. There is very likely no need to sense mechanical deformation with a resolution in the μm range as muscle contraction or relaxation usually involve several millimeters of the gut wall.

The voltage sensitive dye recordings did not reveal receptor potentials generated in specialized trigger zones but rather detected action potentials in an all or none fashion. The failure to record receptor potentials was likely due to the mechanical artifacts at the site where the carbon fiber was pressed on the neurite which hampered the detection of small amplitude potentials, even after signal filtering. In addition according to the literature (Gardner and Martin, 2000) the amplitude of receptor potentials is < 1.5 mV, which makes it difficult to resolve them with the voltage sensitive dye technique.

In a proof of principle study we performed experiments on cultured human myenteric neurons and demonstrated that their mechanosensitive properties are similar to guinea pig myenteric neurons. This reinforces our previous findings that properties of MEN are preserved across different species (Mazzuoli and Schemann, 2012).

Further experiments are required to identify the neuronal structures that encode the mechanical deformation: which channels and/or cytoskeleton modifications encode the mechanical signal and mediate the electrical response. Other experiments are needed to find out whether and how enteric neurons respond to different stimulus modalities or whether specific receptors encode primarily one type of mechanical stimulus.

In conclusion, we provided some novel insights into the response patterns of MEN. We demonstrated that around 50% of cultured enteric neurons possessed mechanosensitive properties. Mechanosensitivity was not restricted to hot spots at terminal endings but was instead located on the soma and along the entire length of the neuronal processes. We showed that mechanosensitive neurites were able to conduct spikes in both directions which suggested that they also serve efferent functions. Finally, we demonstrated that MEN share some properties with other classical sensory neurons but also exhibit specific features very likely reflecting adaptation to their specialized functions in the gut.

## Author contributions

Conceived and designed the experiments: EK, GM, MS. Performed the experiments: EK, GM. Analyzed the data: EK, GM, KM. Contributed reagents/materials/analysis tools: EK, KM, FZ, ID, GC, MS, GM. Wrote the paper: EK, GM, MS, KM.

## Funding

This work is supported by the German Research Foundation DFG (MA-5202/1-1 and 1-2) and the Technische Universität München within the funding program Open Access Publishing.

### Conflict of interest statement

The authors declare that the research was conducted in the absence of any commercial or financial relationships that could be construed as a potential conflict of interest.

## Abbreviations

AH: after-spike hyperpolarization
AI: adaptation index
CCD: charge coupled device
DRG: dorsal root ganglia
ENS: enteric nervous system
ICA: independent component analysis
IPAN: intrinsic primary afferent neuron
LMMP: longitudinal muscle—myenteric plexus
MEN: mechanosensitive enteric neurons
RAMEN: rapidly adapting mechanosensitive enteric neurons
SAMEN: slowly adapting mechanosensitive enteric neurons
TTX: tetrodotoxin
USAMEN: ultra-slowly adapting mechanosensitive enteric neurons.
